# Multiple Preheating Processes for Suppressing Liquefaction Cracks in IN738LC Superalloy Fabricated by Electron Beam Powder Bed Fusion (EB-PBF)

**DOI:** 10.3390/ma17225667

**Published:** 2024-11-20

**Authors:** Yang Li, Hongyu Long, Bo Wei, Jun Zhou, Feng Lin

**Affiliations:** 1 Department of Mechanical Engineering, Tsinghua University, Beijing 100084, China; liyang.2018@tsinghua.org.cn (Y.L.);; 2 Institute of Laser Intelligent Manufacturing and Precision Processing, School of Mechanical Engineering, Guangxi University, Nanning 530004, China

**Keywords:** non-weldable superalloys, EB-PBF, liquefaction crack, multiple preheating process, microstructure, mechanical properties

## Abstract

In additive manufacturing, controlling hot cracking in non-weldable nickel-based superalloys poses a significant challenge for forming complex components. This study introduces a multiple preheating process for the forming surface in electron beam powder bed fusion (EB-PBF), employing a dual-band infrared surface temperature measurement technique instead of the conventional base plate thermocouple method. This new approach reduces the temperature drop during forming, decreasing surface cooling by 28.6% compared to traditional methods. Additionally, the precipitation of carbides and borides is reduced by 38.5% and 80.1%, respectively, lowering the sensitivity to liquefaction cracking. This technique enables crack-free forming at a lower powder bed preheating temperature (1000 °C), thereby improving the powder recycling rate by minimizing powder sintering. Microstructural analysis confirms that this method reduces low-melting eutectic formation and alleviates liquefaction cracking at high-angle grain boundaries caused by thermal cycling. Consequently, crack-free IN738 specimens with high-temperature durability were successfully achieved, providing a promising approach for the EB-PBF fabrication of crack-resistant IN738 components.

## 1. Introduction

Nickel-based high-temperature alloys have been widely used in industrial gas turbines and aerospace engine blades [1,2,3] due to their excellent microstructure stability [4], high-temperature strength [5,6], excellent high-temperature oxidation resistance [7], and creep resistance [8,9]. With the ongoing application and development of these nickel-based superalloys (usually used above 800 °C), higher demands are being placed on their production and fabrication processes, particularly for parts with complex shapes and structures [10]. Currently, additive manufacturing (AM) technology has been successfully used to produce various nickel-based alloys, such as IN625 [11], IN718 [12], IN738 [13,14], IN939 [15], and CMSX-4 [16]. A key focus of research in the AM fabrication of nickel-based high-temperature alloys is how to effectively suppress cracking during the process.

For the weldable IN718 high-temperature alloy, its weak crack sensitivity allows for effective control by adjusting processes, such as scan strategy [17], energy density [18], and other post-processes [19]. In contrast, IN738 contains higher levels of Al and Ti (above 6.7 wt.%) [19,20,21], classifying it as a typical non-weldable superalloy (alloys with Al and Ti contents exceeding 4 wt.% can be categorized as non-weldable). It exhibits significant crack sensitivity [22,23,24]. This presents a challenge for the additive manufacturing (AM) process, as the complex thermal environment created by the cyclic heating and cooling of the melt pool introduces significant stresses that greatly influence the microstructure and properties of the formed part [16,25,26].

Currently, researchers have investigated the liquefaction crack formation mechanism in additively manufactured IN738 superalloy. Xu et al. [27] suggested that the γ + γ′ microstructure forms low-melting-point eutectics and precipitates along grain boundaries (GBs), leading to liquefaction cracks. Ramakrishnan et al. [24] found a large number of low-melting eutectic phases of γ + γ′, carbides, and borides near cracks in IN738. Additionally, Rielli et al. [13] believe that the liquefaction crack is related to the micro-segregation of W, B, and C elements at the grain boundary. Edouard et al. [28] suggested that the formation of liquation cracks requires certain preconditions. The liquation of low-melting-point eutectic phases enriched with B weakens the grain boundary strength, leading to the tearing and cracking of grain boundaries under cyclic thermal stress. However, the thermal stress induced by cyclic heating and cooling during additive manufacturing is not the sole cause of grain boundary tearing. Further evidence indicates that micro-stress at high-angle grain boundaries (HAGBs) and dislocation accumulation also contribute to cracking [26,28]. It has been observed that liquation cracks often occur near high-angle grain boundaries [14], further supporting this viewpoint.

In summary, the liquation of γ + γ′ eutectics precipitated at grain boundaries (GBs) at lower temperatures is the fundamental cause, while the high-frequency thermal cycling ultimately triggers liquation cracking. These factors are closely related to temperature fluctuations during the additive manufacturing process. Therefore, controlling temperature variation presents a new approach to suppress liquefaction cracking. For example, Hagedorn et al. successfully prepared crack-free nickel-based high-temperature alloy M247 by heating the entire forming chamber to 1200 °C using induction heating during EB-PBF [29]. Similarly, Xu et al. [30] successfully limited the propagation of liquefaction cracks by heating the substrate to 1050 °C with medium-frequency induction and by controlling the columnar-to-equiaxed transition (CET), which restricted the growth of the rod-like γ′ phase.

However, current research primarily relies on temperature measurements taken from the printed substrate [5,28,31]. As the build height increases, this method can lead to significant temperature measurement errors. Furthermore, existing preheating studies have not considered the extended cooling intervals left after powder spreading, which evidently cause substantial temperature fluctuations. Moreover, nearly all preheating processes that depend on bottom plate temperature measurements fail to detect these fluctuations, which could explain why unexpected liquation cracks occur in EB-PBF even at higher preheating temperatures. Currently, there is an incomplete understanding and insufficient research regarding the impact of powder bed cooling after powder spreading on liquation cracking in the EB-PBF process.

Therefore, this paper utilizes an infrared dual-band thermometer to monitor surface temperature during the EB-PBF process, enabling accurate tracking of temperature variations at each stage from powder spreading to preheating and forming. The precipitation mechanism of low-melting-point eutectic borides and carbides at crack grain boundaries was systematically investigated. The study explores the control mechanisms of multiple preheating processes on liquation cracking and clarifies the relationship between microalloying element segregation, grain boundary characteristics, and temperature fluctuation magnitude. This approach provides valuable insights for optimizing parameters and adjusting the preheating process. Based on measurement results and existing equipment, a multiple preheating process was developed, effectively mitigating the sudden drops in surface temperature caused by printing and powder spreading stages, achieving the goal of printing IN738 superalloys without liquation cracks at 1000 °C.

## 2. Materials and Methods

### 2.1. The Multiple Preheating Process

**Figure 1:**
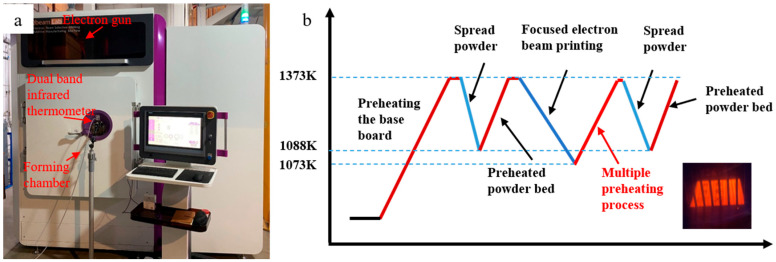
EB-PBF multiple preheating process (**a**) EB-PBF equipment based on the multiple preheating process. (**b**) Schematic diagram of the EB-PBF process utilizing the multiple preheating method (the slope of the broken line is illustrative only and does not represent the actual heating rate).

It is well known that high-temperature preheating (500 °C to 1200 °C) is a key advantage and feature of EB-PBF technology. However, the low-power focused electron beam used for forming, along with the subsequent powder lay-up process, causes a sudden temperature drop on the forming surface. The conventional method of measuring temperature using base plate thermocouples cannot effectively monitor these temperature fluctuations in real-time, leading to the formation and propagation of thermal cracks. To address this issue, the surface temperature of the formed part is monitored online through the chamber window using a dual-band infrared thermometer (Figure 1a). Additionally, a new multiple preheating process has been developed. Electrons generated by the cathode in the electron gun are accelerated by a high-voltage (60 kV) electric field between the cathode and anode to form an electron beam. The focus coil controls the electron beam’s focal distance and size, while the deflection coil ensures precise targeting of the electron beam to the desired position on the forming surface for scanning. By adjusting the electron beam’s focal length and size, the preheating process and parameters can be easily optimized. To mitigate the temperature drop caused by powder spreading, a multiple preheating process is proposed. As shown in Figure 1b, before depositing each layer of powder, the powder bed is preheated multiple times by gradually increasing the power of the defocused electron beam. This preheating method occurs after the focused electron beam deposition is completed and effectively reduces the temperature drop during the printing and powder spreading processes, thereby minimizing temperature fluctuations within the powder bed. The surface temperature is monitored in real-time using an IGAR 12LO dual-band infrared thermometer from LUMASENSE Group, Berlin, Germany. This pyrometer has a temperature range of 500–2200 °C, with temperature bands of 1.28 and 1.65 μm, and a minimum spot size of 1.1 mm. The pyrometer is calibrated using a DYHT3 blackbody radiation source (Taian Demei Mechanical and Electrical Equipment Co., Ltd, Taian, China.) prior to use, ensuring the accuracy of temperature measurements.

### 2.2. Samples Fabrication

In this experiment, pre-alloyed IN738LC powder, prepared by the vacuum induction melting (VIM) aerosolization method by the China Iron and Steel Research Institute Group, was used. The powder has an average particle size of approximately 66 μm, and its microstructure can be referenced in previous studies [6]. The chemical composition of the powder is provided in Table 1.

**Figure 2:**
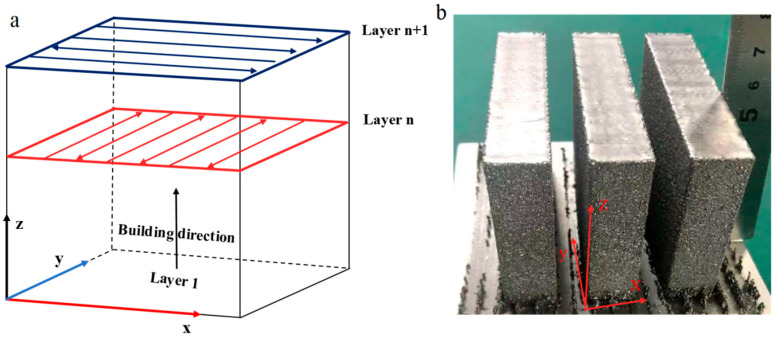
(**a**) The scanning strategy employed for fabricating the studied IN738LC. (**b**) Pictures of samples prepared based on the multiple preheating process.

The specimens for this study were prepared using the EB-PBF equipment (QBeamLab 200) provided by Tianjin QBEAM Technology Co., Tianjin, China. The electron gun acceleration voltage was set to 60 kV. A 10 mm thick 304 stainless steel plate was used as the substrate, and it was preheated using a scattered-focus electron beam (scanning speed v = 20 m/s) to reach a temperature of 1070 °C. Afterward, the powder was spread, and the powder bed was preheated again using a scattered-focus electron beam (v = 27 m/s). For melting the samples, the line offset was set to 0.1 mm, the surface energy density (SED) was varied from 4.2 to 7.0 J/mm^2^, and the scanning speed v ranged from 0.5 to 6.0 m/s. The layer thickness was set at 70 μm. As shown in Figure 2a, the forming scan was performed by rotating the scanning direction by 90° for the *n* + 1th layer after constructing the nth layer. The size of the rectangular specimen was 18 × 70 × 60 mm^3^, as illustrated in Figure 2b. In preliminary experiments, we tested three different preheating temperatures (1000 °C, 1030 °C, and 1060 °C) based on the conventional preheating process for the powder bed (using bottom plate measurements) to investigate the effect of powder bed temperature on cracking and sintering during the formation of IN738 alloy using EB-PBF, while also considering powder recyclability. See Section 3.1 for details. After evaluating the number of cracks in the sample against the economics of powder recycling, we determined that a preheat temperature of 1000 °C was optimal, followed by printing with a focused electron beam (v = 1.5 m/s). After each layer was constructed, we preheated the forming surface again with a scattered-focus electron beam, with specific preheating parameters outlined in Table 2.

### 2.3. Microstructure Characterization

All samples were obtained through EDM wire cutting. The samples were initially polished using sandpaper, followed by chemical etching (10 HCl:1 HNO_3_:10 H_2_O, 7 min) to prepare them for Keyence VHX-7000 optical microscope (OM) and scanning electron microscopy (SEM) analysis. The tilt angle of the SEM carrier table, θ, ranged from 0° to 15°, facilitating the observation of crack morphology within the material. The elemental distribution near the cracks was examined using energy-dispersive spectroscopy (EDS). The EBSD samples were initially prepared through mechanical polishing, using sandpapers with grit sizes of 200 #, 800 #, 1200 #, and 2000 # in sequence. They were then polished with a 50 nm SiO_2_ suspension and finally electrochemically polished at room temperature (7 HClO_4_:93 C_2_H_6_O, 30 V, 1 min). EBSD data analysis was performed using OIM v5.0 software from EDAX Inc. To further investigate elemental micro-polarization behavior, an electron probe microanalyzer (EPMA) was used. For transmission electron microscopy (TEM) sample preparation, initial thinning was achieved by mechanical polishing followed by manual grinding with incrementally finer sandpaper until the sample thickness reached approximately 200 μm. The samples were further thinned to about 50 μm using a dimple grinder, and surface scratches were removed with a polisher. Finally, focused ion beam (FIB) milling to a thickness of 50–100 nm. The types of precipitated phases were determined by TEM observation at preferentially selected elemental deviations, with an EDS probe for further analysis. Finally, EBSD data were analyzed to explore the relationship between cracking and misorientation angles, where low-angle grain boundaries (LAGBs) are defined by misorientation angles (θ_ma_) between 2 and 15° and high-angle grain boundaries (HAGBs) by angles greater than 15°.

### 2.4. High-Temperature Endurance Strength Test

**Figure 3:**
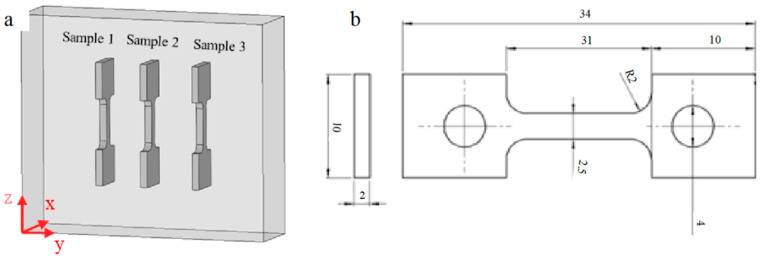
Schematic of the high-temperature endurance strength test specimens. (**a**) The locations in the as-built sample, and (**b**) the dimensions of the high-temperature enduring strength test specimens.

For the IN738 nickel-based high-temperature alloy, measuring its high-temperature creep strength is crucial as a basis for selecting materials for the design of high-temperature components. To achieve this, a continuous uniaxial tensile creep test was conducted to determine the time to creep fracture and the creep elongation of samples prepared using the multiple preheating process. These samples were subjected to a constant load at a specified temperature, and the cross-sections of the fractured samples were observed using SEM. Small plate-shaped specimens were cut by EDM, as shown in Figure 3. The specimen axis was aligned parallel to the build direction (BD) of the EB-PBF process. The test temperature was set at 850 °C, the applied stress was at 365 MPa, and the testing standard followed the national standard GB/T 2039-2012 [32] of the People’s Republic of China.

## 3. Results and Discussion

### 3.1. Effect of Preheating Temperature on Crack and Powder Sintering

**Figure 4:**
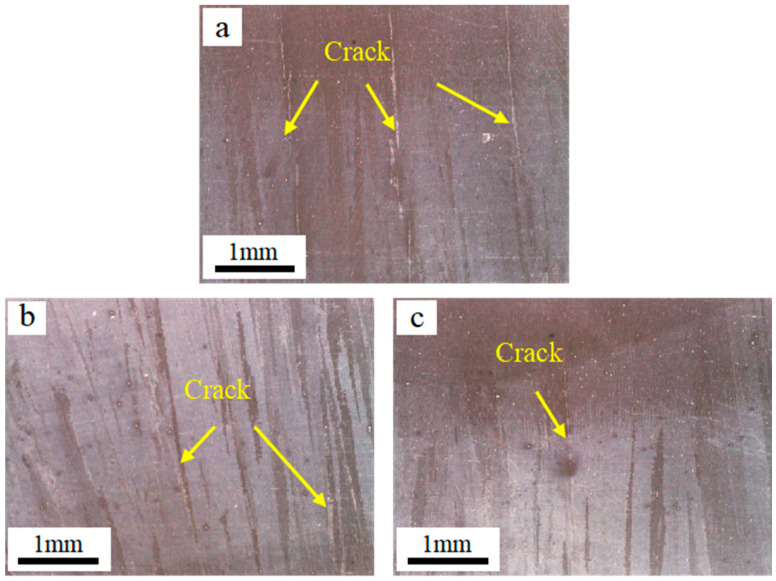
The macroscopic cracks of the sample under different preheating temperatures of the substrate are (**a**) 1000 °C, (**b**) 1030 °C, and (**c**) 1060 °C.

The formation of cracks in IN738 alloy is highly sensitive to temperature changes. Therefore, we opted to study it under the smallest possible temperature gradients. The internal cracks in specimens prepared at different substrate preheating temperatures (1000 °C, 1030 °C, and 1060 °C) were observed using an optical microscope (OM) manufactured by Ningbo Yongxin Optics Co., Ltd. (Ningbo, China), as shown in Figure 4. The figure shows a significant reduction in both the number and length of internal cracks as the preheating temperature increases. This suggests that the initiation of internal cracks can be minimized by raising the preheating temperature of the substrate. Consequently, the cracking tendency in IN738 alloy formed by EB-PBF can be mitigated to some extent by increasing the preheating temperature.

**Figure 5:**
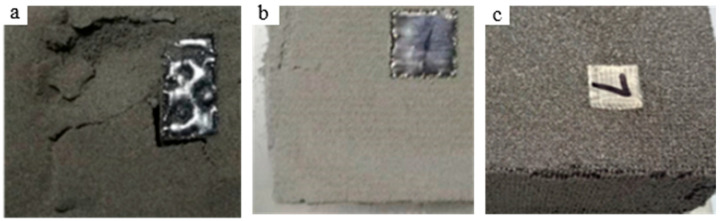
Powder bed under different preheating temperatures (**a**) T_Sub1_ = 1000 °C, (**b**) T_Sub2_ = 1030 °C, and (**c**) T_Sub3_ = 1060 °C.

However, in the EB-PBF process, reducing the preheating temperature of the base plate appropriately can prevent excessive sintering of the powder bed. This enhances the sustainability of powder usage and brings significant economic benefits. On the one hand, only by increasing the preheating time can the powder bed temperature be maintained at 1060 °C or higher, which would reduce printing efficiency. On the other hand, excessively high preheating temperatures cause severe powder sintering, making it difficult to recycle, which increases costs. As shown in Figure 5a, the powder bed preheated at 1000 °C can be easily crushed by hand, indicating that it remains loose and easy to recycle. When the preheating temperature was increased to 1030 °C, the powder bed surface became flatter and harder, though the edges were still crushable (Figure 5b). However, as shown in Figure 5c, when the preheating temperature reached 1060 °C, the powder bed sintered severely, resulting in a dense, hard surface that was difficult to remove with sand blowers, making powder recycling and reuse challenging, which leads to unnecessary waste.

### 3.2. Real-Time Detection of Surface Temperature Based on Dual-Band Infrared Thermometer

**Figure 6:**
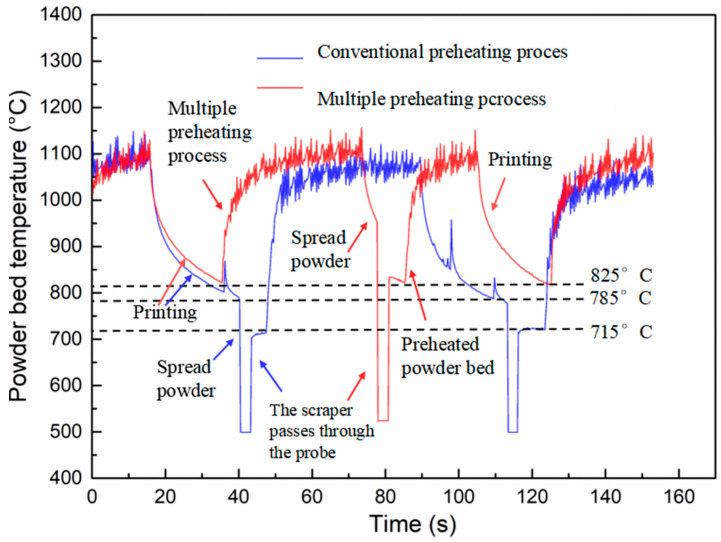
Comparison of surface temperature between multiple preheating processes and conventional process.

To address the issue of powder bed recyclability while also eliminating cracks caused by insufficient preheating, we developed a multiple preheating process for the powder bed based on existing equipment. The surface temperature of the multiple preheating process was compared with that of the conventional preheating process using a dual-band infrared thermometer at a substrate preheating temperature of 1000 °C, as shown in Figure 6. In the conventional preheating process, scattered preheating is stopped and switched to focused forming mode at each layer of the forming scan, which reduces the heated surface area. This results in a significant drop in surface temperature, from 1100 °C to approximately 800 °C. At the end of each layer-forming scan, the powder bed temperature further decreases from 800 °C to around 715 °C due to the lower temperature of the newly spread powder. The powder bed undergoes an initial temperature drop during the forming process, followed by a second drop during powder spreading. These two significant temperature fluctuations can create large temperature gradients and thermal stress, increasing the sensitivity to cracking. It is important to note that the temperature measurement device, an infrared thermometer, measures the surface temperature of the powder brush during the powder spreading process. During powder spreading, the powder bed temperature value momentarily drops to 500 °C below the measurement line, but it returns to normal after the powder brush has passed. The red curve represents the surface temperature change in the multiple preheating process. As shown, the post-preheat process raises the powder bed temperature of the printing surface back to 1100 °C, and after the powder brush spreads the powder, the powder bed temperature only drops to around 825 °C.

After comparing these two preheating processes, we found that the multiple preheating process reduces surface temperature fluctuations by approximately 110 °C, as shown in Figure 6. This reduction in temperature gradient during the printing process helps to lower thermal stress, thereby mitigating the tendency for crack initiation.

**Figure 7:**
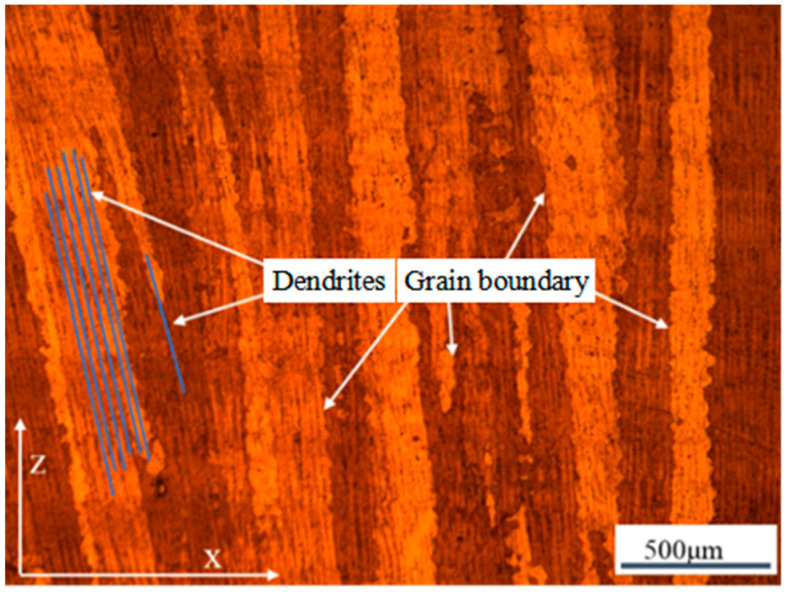
Images of the formed samples without cracks prepared by the multiple preheating process.

By using the multiple preheating process, crack-free forming can be achieved at a powder bed preheating temperature of 1000 °C. This process not only enables the crack-free forming of difficult-to-weld high-temperature alloys but also ensures that the powder can be recycled at a lower powder bed temperature. Figure 7 shows optical microscope images of the internal structure of the crack-free specimen. The microstructure is primarily composed of columnar crystals, which gradually transition into dendritic crystals as the build height increases, with no cracks observed throughout the structure.

### 3.3. Liquefaction Crack Characterization

It is well known that cracking during the EB-PBF process results from the competition between the driving forces of cracking—namely, the stress and strain generated by rapid cyclic heating and cooling—and the grain boundary strength. The strength of these grain boundaries is further weakened by the localized liquefaction of the γ-phase, leading to cracking along the grain boundaries under stress. Another known cause of cracking in the EB-PBF-IN738 alloy is the large tensile stress caused by the rapid precipitation of γ′ particles [30]. From the thermal cracking control process discussed in the previous section, it is evident that the multiple preheating process effectively reduces susceptibility to liquefaction cracking by minimizing temperature fluctuations on the forming surface, reducing the thermal gradient and solidification rate, and lowering the γ′ phase precipitation rate to reduce shrinkage stress [21]. To further investigate the types and initiation mechanisms of cracks during the EB-PBF preparation of IN738LC, the cracks were systematically characterized in this study.

**Figure 8:**
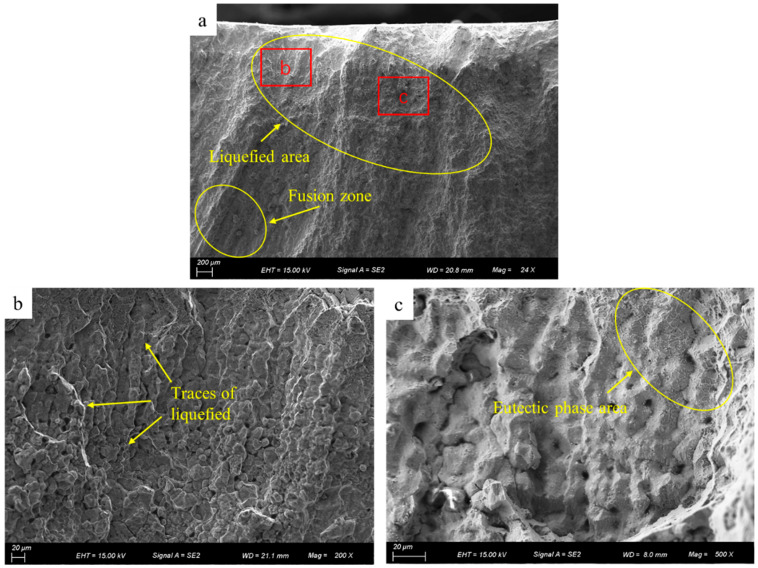
Direct observation of the side by SEM (**a**) liquefaction area, (**b**) liquefaction trace in (**a**), and (**c**) eutectic phase area.

As shown in Figure 8, the fracture side with cracks was observed using SEM. The low-magnification image of the fracture in Figure 8a reveals a large, liquefied area above the fusion zone. The crack fracture in Figure 8b shows clear signs of surface liquefaction. The typical dendritic structure of the fracture surface can be clearly seen in Figure 8c, indicating that a continuous liquid film covers almost the entire fracture cross-section of the sample. The white mesh marked by the yellow circle represents the low-melting-point eutectic precipitation phase. This enriched region is consistent with the low-melting-point eutectic phase observed by Zhang et al. [24] during the preparation of IN738LC alloy by laser melting deposition. Therefore, the crack is tentatively identified as a liquefaction crack.

**Figure 9:**
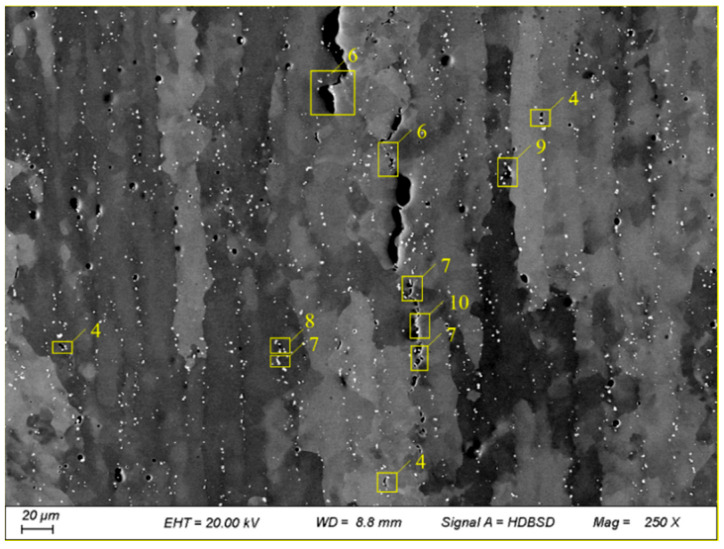
Carbide segregation at grain boundary near the crack (The numbers in the figure represent the quantity of carbides).

The compositional alignment near the crack was observed using SEM in backscatter mode, as shown in Figure 9. The yellow box highlights some of the carbides, with the number of carbide particles marked. From the figure, it is evident that there is a clear tendency for carbides to precipitate at the crack and dendrite boundaries. Additionally, there is a significant increase in the number of carbide particles within the yellow boxes, which aligns with the observation by Xu et al. that low-melting-point eutectics tend to precipitate at grain boundaries (GBs), leading to cracking [27].

**Figure 10:**
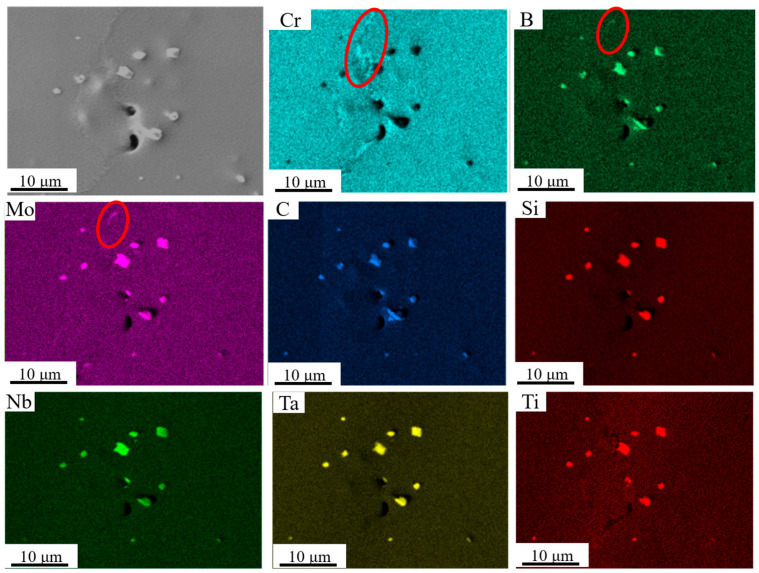
EDS surface scan results of the area near the crack.

To further investigate the effect of elemental distribution on liquefaction cracks, an EDS mapping analysis was conducted on the area near the crack in Figure 9, as shown in Figure 10. The results indicate that carbides containing Mo, B, Ti, Ta, Nb, and Si are enriched near the crack. Additionally, closer observation reveals precipitates of Mo, B, and Cr near the grain boundaries of the crack. Edouard et al. similarly found boride precipitate phases, such as MB, M_2_B, and Cr_5_B_3_, enriched with Mo and Cr near liquefaction cracks during EB-PBF preparation of refractory nickel-based high-temperature alloys [28]. Montazeri et al. also observed similar low-melting-point borides enriched with Mo and Cr near liquefaction cracks during laser welding of the IN738 high-temperature alloy [33]. Furthermore, Zhong et al. [34] found numerous carbides containing Ti, Nb, and Ta precipitated near cracks in the IN738 direct energy deposition process. This provides further evidence that the low-melting-point eutectic precipitation phase is the root cause of liquefaction cracks.

**Figure 11:**
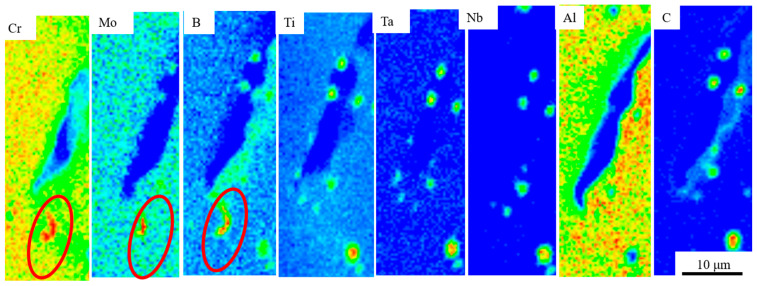
EMPA surface scanning analysis of crack samples.

To more clearly and accurately distinguish carbides from liquefied precipitates in the eutectic phase, this study conducted an EMPA scan of the cracked sample, with the results shown in Figure 11. The figure reveals that carbides containing B, Ti, Ta, and Nb elements precipitated at dendritic boundaries near the cracks and grain boundaries. These are typical MC-type carbides found in IN738LC alloy [35], but no M23C6-type carbides were detected. Studies have shown that MC-type carbides are prone to bulk liquefaction mechanisms, which can lead to liquefaction cracking [13]. They are usually formed by local melting at the second-phase particle/matrix interface under the non-equilibrium conditions of the rapid heating associated with additive manufacturing [36].

It is worth noting that, in comparison with Figure 9, Mo and Si showed overlapping data with C and Si in the EDS test spectra. However, since Si and C content are closely related to solidification cracking [37], it may not be fully accurate to judge the presence of carbides based solely on EDS results. As indicated by the red-circled region in Figure 11, low-melting-point eutectic borides enriched in Mo and Cr precipitate at grain boundaries near the cracks.

**Figure 12:**
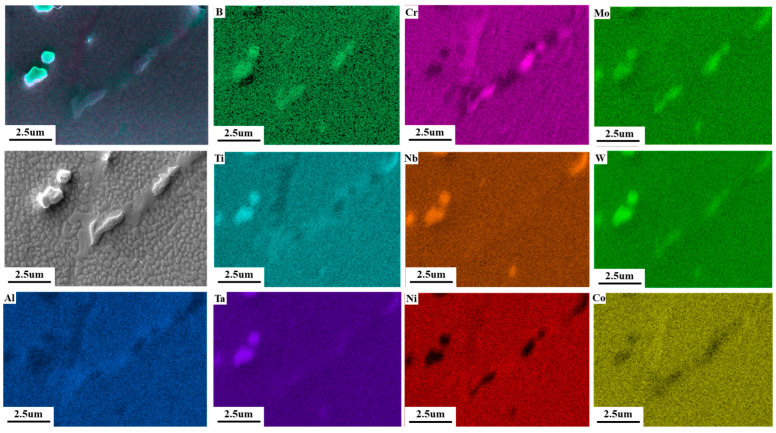
EDS surface scan analysis of FIB positioning segregation phase.

**Figure 13:**
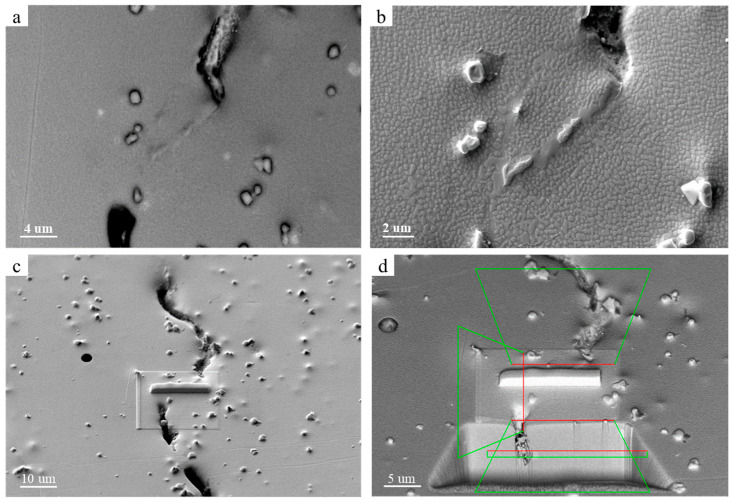
SEM observation of the B-rich area: (**a**) 3.08 K magnification image, (**b**) 10.89 K magnification image, (**c**) marking of cutting position, and (**d**) positioning of cutting.

To accurately calibrate the B-rich phase, we continued sampling using focused ion beam technology. The B-enriched region was first identified under SEM observation, as shown in Figure 12. The selected area was then coated with a layer of metallic platinum for protection and subsequently cut using the focused ion beam. The entire cutting process is illustrated in Figure 13a–d.

**Figure 14:**
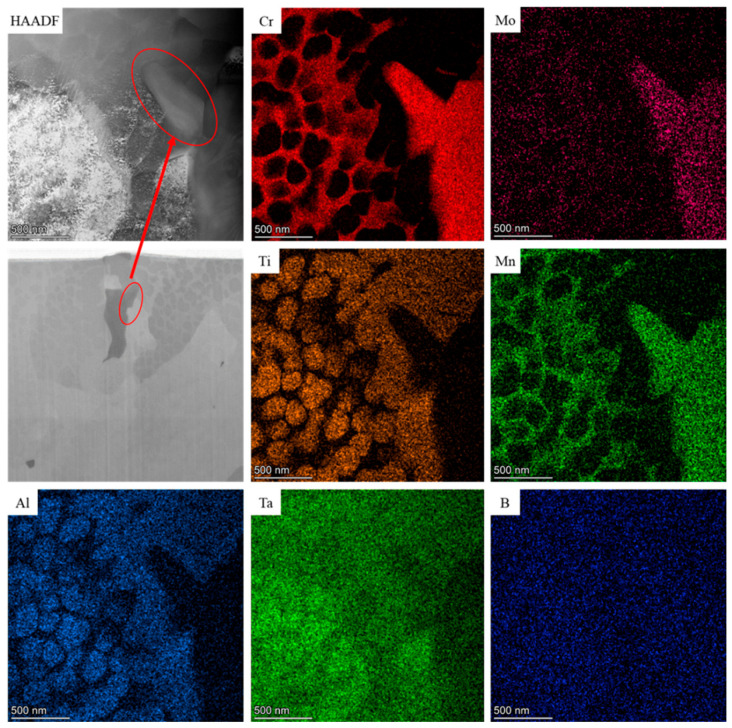
TEM spectral surface scanning analysis of FIB cut samples.

**Figure 15:**
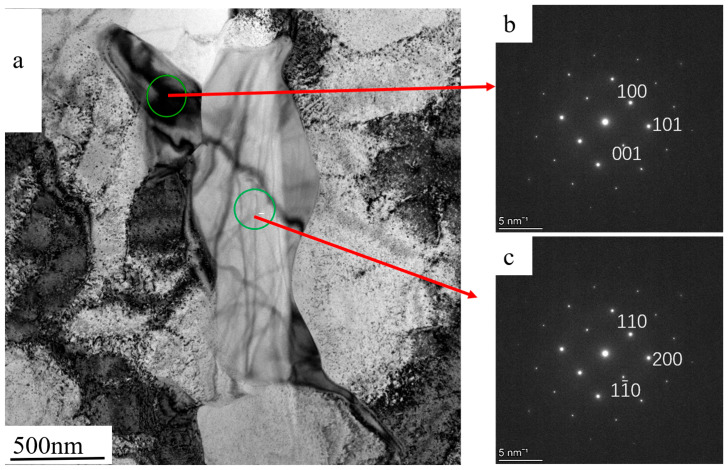
Trailing phase TEM bright field and electron diffraction pattern in FIB cut sample (**a**) TEM bright field morphology, (**b**) upper SAED calibration, (**c**) lower SAED calibration.

As shown in Figure 14, the FIB-cut sample contains a tail-shaped phase, and energy spectrum analysis of this phase indicates the presence of Cr and Mo elements. TEM observation of this phase is shown in Figure 15a. Combined with the elemental analysis from Figure 14, two adjacent precipitated phases of the same composition are present at this location. The SAED (selected area electron diffraction) calibration of the upper black branching phase is shown in Figure 15b. Based on comparison with PDF card 65-7804, this phase is identified as B_2_Cr, where the atomic spacings on the (001), (100), and (101) crystal planes are 0.3066 nm, 0.25712 nm, and 0.19701 nm, respectively.

SAED calibration of the lower phase is shown in Figure 15c. The measured atomic spacings for the (110), (1–10), and (200) crystal planes are 0.37972 nm, 0.37972 nm, and 0.2685 nm, respectively, which, when compared with PDF card 65-2787, confirms that the phase is B_3_Cr_5_. In this phase, Mo and Mn atoms are partially replaced by Cr atoms, forming a substitutional solid solution in the precipitated phase.

### 3.4. High-Temperature Durability

IN738 is a nickel-based high-temperature alloy that is widely used in industrial gas turbines and aero-engine blades, where it must perform in high-temperature environments and withstand significant loads without fracturing. Therefore, in this study, three samples prepared using the multiple preheating process were tested for high-temperature durability under constant temperature and load conditions. This was done to evaluate the impact of liquefaction cracks on the high-temperature durability performance of the samples. The test results of this study are presented in Table 3.

**Figure 16:**
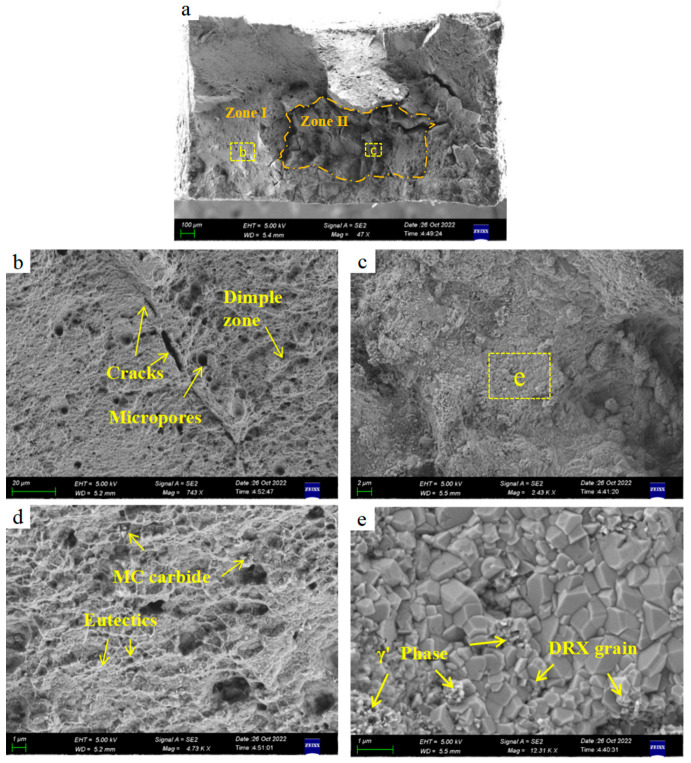
SEM image of fracture surface with high-temperature durability of IN738 sample without crack (**a**) macroscopic structure of fracture; (**b**) intergranular creep failure; (**c**) ductile creep fracture zone; (**d**) SEM high-magnification image of the typical region in Figure 17b; (**e**) high-magnification SEM image of a ductile fracture.

According to the test results in Table 3, all three specimens were prepared using the multiple preheating process and demonstrated excellent high-temperature creep resistance, with each specimen enduring for at least 53 h and up to 69.25 h at 850 °C and 365 MPa without fracture. Additionally, fracture analysis revealed that the high-temperature creep process was accompanied by dynamic recrystallization (DRX) [38], which effectively refined the grains (Figure 16c). While finer grains in polycrystalline materials typically reduce creep resistance at high temperatures due to the increased grain boundary area, all three samples maintained high elongation, up to 24.7%, which is comparable to that of cast IN738.

### 3.5. High-Temperature Creep Fracture Mechanism

The fracture surface of sample No. 1, which successfully completed the high-temperature creep test, was observed using SEM. The microstructure of the fracture surface is displayed in Figure 16.

From the perspective of fracture mechanics, the fracture behavior of metallic materials is determined by the combined effects of internal damage/toughening and external toughening. For additively manufactured IN738 high-temperature alloys, internal damage includes thermal cracks caused by the segregation of second-phase particles such as borides and carbides at grain boundaries. The intrinsic toughening mechanisms mainly involve inhibiting damage behavior, for example, through grain boundary engineering or by introducing microcracks to disperse crack stress. In contrast, external toughening mechanisms, such as the precipitation of a large number of nano-sized γ′ phases, effectively strengthen the matrix, causing cracks to branch and deflect, thereby preventing intergranular fracture.

As the test time increased, the load-bearing area of the sample decreased, leading to a rapid rise in stress. Region II exhibited a completely different fracture morphology, transitioning from the flat fracture surface of Region I to an irregular, raised appearance, with noticeable macroscopic cracks (Figure 16a). The microstructure in Region II changed due to creep behavior; no micropores or networked γ/γ′ eutectic phases were observed, while a significant amount of typical blocky nano γ′ phases precipitated (Figure 16e).

### 3.6. Mechanism Analysis of Liquefaction Crack

**Figure 17:**
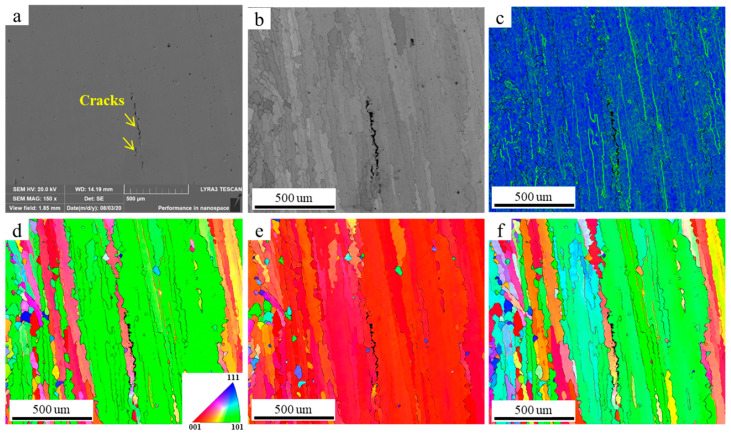
SEM and EBSD maps (obtained using a step size of 2.8 µm) showing the crack along the coarse columnar grain boundary. (**a**) SEM diagram, (**b**) grain boundary (GB) map, (**c**) KAM map, (**d**) IPF X, (**e**) IPF Y, and (**f**) IPF Z.

Existing studies suggest that the formation of liquefaction cracks in additively manufactured refractory nickel-based high-temperature alloys is primarily due to two factors. First, the segregation of B elements at grain boundaries promotes the formation of low-melting-point brittle γ + γ′ phases at the grain boundaries. Second, the melting of the low-melting-point eutectic phase under cyclic heat leads to the formation of a liquid film, which reduces the tensile strength at grain boundaries, causing cracks under thermal stress. Additionally, liquefaction cracking is often observed near large-angle grain boundaries in the heat-affected zone (HAZ), as shown in Figure 17. This occurs because the eutectic phases segregate at the grain boundaries, and when the grain boundary temperature falls below the melting point of the eutectic phase, the more pronounced stress concentration at the HAGBs can cause the liquid film to crack, leading to liquation cracks at the HAGBs.

To further analyze the relationship between liquefaction cracks and grain boundary angles, electron backscatter diffraction (EBSD) analysis was conducted on the region near the crack, as shown in Figure 17. In Figure 17a, the thin red line represents low-angle grain boundaries (2–15°), while the thin black line indicates high-angle grain boundaries (>15°). It can be observed that the crack is located along a high-angle grain boundary, with the crack (black area in Figure 17b) propagating directionally along the grain boundary. Figure 17c displays the Kernel Average Misorientation (KAM) map of the selected region, which shows a pronounced stress concentration at the grain boundary near the crack (indicated by the yellow circle). This further confirms the tendency of liquefaction cracks to initiate at high-angle grain boundaries (HAGBs).

The presence of continuous eutectic liquid films at grain boundaries is a key factor in the formation of liquefaction cracks, as the wetting of grain boundaries by the liquid phase significantly influences cracking behavior. High-angle grain boundaries (HAGBs), which have higher grain boundary energy [39], are more susceptible to wetting by liquid phase penetration [40]. Additionally, the level of B element segregation depends on the grain boundary’s interfacial energy. Reducing temperature fluctuations can suppress the growth of liquid films and bulk liquefaction phenomena. Furthermore, minimizing high-temperature gradients helps prevent the transformation of low-angle grain boundaries (LAGBs) into HAGBs, thereby inhibiting crack propagation at HAGBs.

**Figure 18:**
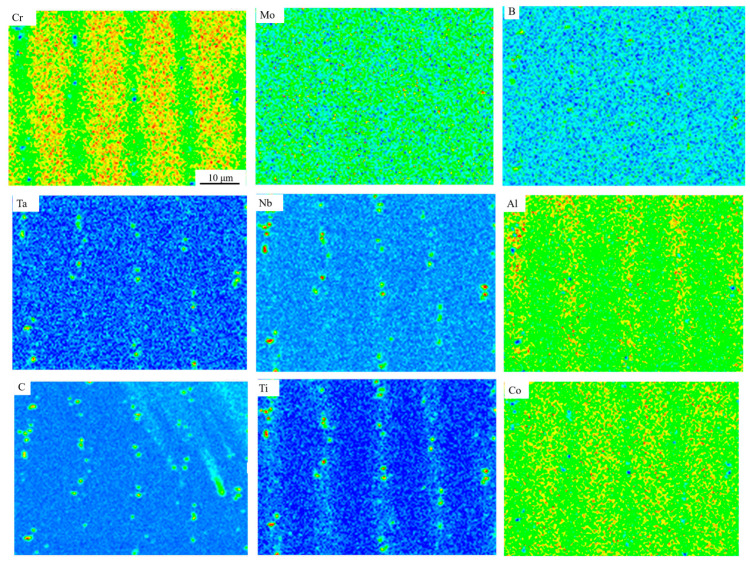
EPMA surface scanning analysis of samples without cracks.

The comparison of surface temperatures between the multiple preheating process and the conventional preheating process, as shown in Figure 6, reveals that the multiple preheating process reduces surface temperature fluctuations and decreases thermal stress caused by temperature gradients. This is the primary advantage of the multiple preheating process. Additionally, when comparing the EPMA elemental segregation in the crack-free sample prepared using multiple preheating (Figure 18) with the sample in Figure 11, it is evident that the crack-free sample exhibits a significantly reduced amount of low-melting-point eutectic borides and carbides. Elements such as B and Cr do not accumulate at the grain boundaries, reducing the susceptibility of these boundaries to cracking.

**Figure 19:**
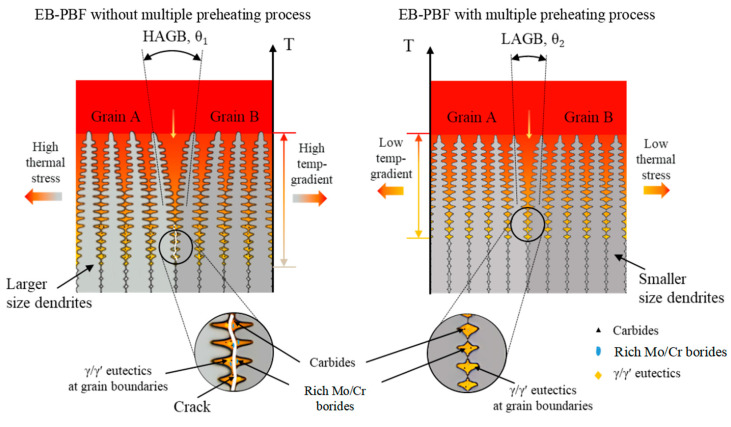
Mechanism of liquefaction crack.

**Figure 20:**
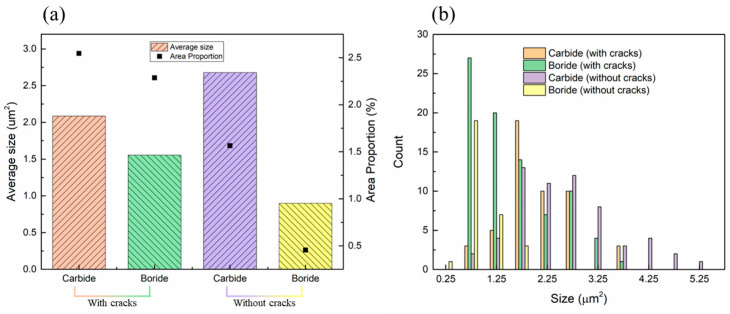
Average size and distribution curves of carbides and borides in cracked and uncracked samples. (**a**) Average size and (**b**) distribution curves.

In summary, as shown in Figure 19, the multiple preheating process reduces temperature fluctuations and thermal stresses while effectively preventing the precipitation of carbides and low-melting-point borides near grain boundaries caused by complex thermal cycles. As shown in Figure 20, the implementation of the multiple preheating process resulted in significant changes in the average size and particle size distribution curves of carbides and borides between samples with cracks and those without. In the crack-free samples, the proportions of carbide and boride precipitates were 1.56% and 0.45%, respectively, representing reductions of 38.5% and 80.1% compared to the samples with cracks. However, the size of the carbides slightly increased. That suppresses the liquefaction of grain boundaries at high temperatures and reduces susceptibility to liquefaction cracks. By addressing these factors, the multiple preheating process successfully suppressed liquefaction cracks in the IN738 samples formed using the EB-PBF method.

## 4. Conclusions

A multiple preheating process based on dual-band infrared surface thermometry was developed to successfully control the tendency of liquefaction cracks to form. The multiple preheating process was compared with the conventional preheating process, and the prepared samples were characterized in terms of microstructure and mechanical properties. The following conclusions were drawn:

The multiple preheating process reduces the temperature drop on the forming surface by about 110 °C, lowering the temperature gradient and reducing thermal stress.

The multiple preheating process allows for a lower powder bed preheating temperature, effectively preventing powder bed sintering and improving the powder recovery rate.

The multiple preheating process inhibits the precipitation of low-melting-point γ/γ′ eutectics at grain boundaries and alleviates the liquefaction of eutectic precipitates during complex thermal cycling.

The precipitation of carbides and borides is reduced by 38.5% and 80.1%, respectively, and the sensitivity of the liquefaction crack is reduced.

With optimized printing parameters, the multiple preheating process enables the printing of crack-free IN738LC high-temperature alloys at a lower powder bed preheating temperature (1000 °C).

IN738LC samples prepared using the multiple preheating process exhibited excellent high-temperature creep resistance, with cross-sectional analysis revealing a mixed creep failure mode.

Although the multiple preheating processes in this article can reduce printing cracks in complex parts, the multiple preheating strategies lower forming efficiency. This approach is only suitable for special cases where conventional methods cannot address the challenges of costly, difficult-to-process materials.

## Figures and Tables

**Table 1:** IN738LC alloy powder composition (wt.%).

Element	Ni	Ti	Al	Co	Cr	C	Fe	Zr	Nb	Ta	Mo	Si	B	W	Mn
wt.%	Bal.	3.39	3.61	8.57	15.92	0.11	0.16	0.019	0.93	1.85	1.86	0.038	0.0077	2.75	0.013

**Table 2:** Preheating parameters of IN738 alloy powder bed.

The Process Parameters	Parameter Settings
Substrate temperature, T_p_ (°C)	1000
Scanning speed, V (m/s)	10–30
Focusing on the current, I_f_ (mA)	(−60) to 60
Scanning electron beam current, I_s_ (mA)	15–40
Preheating times, t (s)	1–2–30
Multiple preheating times	40

**Table 3:** High-temperature durability of three samples at 850 °C/365 MPa.

The Sample	Temperature (°C)	Stress (MPa)	Duration of Time (h)	Elongation (A%)
1	850	365	69.25	12.9
2	850	365	65.05	24.70
3	850	365	53.30	12.7

## Data Availability

The original contributions presented in the study are included in the article, further inquiries can be directed to the corresponding authors.

## Nomenclature

Name	Definition
EB-PBF	Electron beam powder bed fusion
L-PBF	Laser powder bed fusion
GB	Grain boundary
HAGB	High-angle grain boundary
LAGB	Low-angle grain boundary
CET	Columnar-to-equiaxed transition
VIM	Vacuum induction melting
EDM	Electric discharge machining
OM	Optical microscope
EDS	Energy-dispersive spectroscopy
SEM	Scanning electron microscopy
TEM	Transmission electron microscopy
EBSD	Electron backscatter diffraction
SAED	Selected area electron diffraction
EPMA	Electron probe microanalyzer
FIB	Focused ion beam
BD	Building direction
DRX	Dynamic recrystallization
HAZ	Heat-affected zone
KAM	Kernel average misorientation
SED (J/mm^2^)	Surface energy density
V (m/s)	The scanning speed of the electron beam
T_Sub_	The temperature of the substrate
T_bed_	The temperature of the powder bed
I_f_ (mA)	Focusing on the current
I_s_ (mA)	Scanning electron beam current
T (s)	Preheating times
Θ (°)	The tilt angle of the SEM carrier table
θ_MA_ (°)	Misorientation angles
